# Functional lung imaging of 2-year-old children after congenital diaphragmatic hernia repair using dynamic mode decomposition MRI

**DOI:** 10.1007/s00330-023-10335-6

**Published:** 2023-11-08

**Authors:** Efe Ilicak, Greta Thater, Safa Ozdemir, Jascha Zapp, Lothar R. Schad, Stefan O. Schoenberg, Frank G. Zöllner, Meike Weis

**Affiliations:** 1 Computer Assisted Clinical Medicine, Medical Faculty Mannheim, Heidelberg University, Mannheim, Germany; 2 Mannheim Institute for Intelligent Systems in Medicine, Medical Faculty Mannheim, Heidelberg University, Mannheim, Germany; 3 Department of Clinical Radiology and Nuclear Medicine, Medical Faculty Mannheim, Heidelberg University, Mannheim, Germany

**Keywords:** Congenital diaphragmatic hernia, Pediatrics, Functional MRI, Perfusion magnetic resonance imaging, Ventilation perfusion ratios

## Abstract

**Objectives:**

To investigate the feasibility of non-contrast-enhanced functional lung imaging in 2-year-old children after congenital diaphragmatic hernia (CDH) repair.

**Methods:**

Fifteen patients after CDH repair were examined using non-contrast-enhanced dynamic magnetic resonance imaging (MRI). For imaging two protocols were used during free-breathing: Protocol A with high temporal resolution and Protocol B with high spatial resolution. The dynamic images were then analysed through a recently developed post-processing method called dynamic mode decomposition (DMD) to obtain ventilation and perfusion maps. The ventilation ratios (V_Ratio_) and perfusion ratios (Q_Ratio_) of ipsilateral to contralateral lung were compared to evaluate functional differences. Lastly, DMD MRI-based perfusion results were compared with perfusion parameters obtained using dynamic contrast-enhanced (DCE) MRI to assess agreement between methods.

**Results:**

Both imaging protocols successfully generated pulmonary ventilation (V) and perfusion (Q) maps in all patients. Overall, the V_Ratio_ and Q_Ratio_ values were 0.84 ± 0.19 and 0.70 ± 0.24 for Protocol A, and 0.88 ± 0.18 and 0.72 ± 0.23 for Protocol B, indicating reduced ventilation ($p<0.05$) and perfusion ($p<0.01$) on the ipsilateral side. Moreover, there is a very strong positive correlation ($r>0.89,p<0.01$) and close agreement between DMD MRI-based perfusion values and DCE MRI-based perfusion parameters.

**Conclusions:**

DMD MRI can obtain pulmonary functional information in 2-year-old CDH patients. The results obtained with DMD MRI correlate with DCE MRI, without the need for ionising radiation or exposure to contrast agents. While further studies with larger cohorts are warranted, DMD MRI is a promising option for functional lung imaging in CDH patients.

**Clinical relevance statement:**

We demonstrate that pulmonary ventilation and perfusion information can be obtained in 2-year-old patients after CDH repair, without the need for ionising radiation or contrast agents by utilising non-contrast-enhanced MRI acquisitions together with dynamic mode decomposition analysis.

**Key Points:**

*• Non-contrast-enhanced functional MR imaging is a promising option for functional lung imaging in 2-year-old children after congenital diaphragmatic hernia.*

*• DMD MRI can generate pulmonary ventilation and perfusion maps from free-breathing dynamic acquisitions without the need for ionising radiation or contrast agents.*

*• Lung perfusion parameters obtained with DMD MRI correlate with perfusion parameters obtained using dynamic contrast-enhanced MRI.*

**Supplementary information:**

The online version contains supplementary material available at 10.1007/s00330-023-10335-6.

## Introduction

Congenital diaphragmatic hernia (CDH) belongs to rare diseases and is caused by a developmental defect, which occurs in 1 of 2000–5000 live births [1]. CDH is characterised by herniation of abdominal organs into the thoracic cavity and results in impaired lung development, followed by irreversible hypoplasia of the pulmonary parenchyma and vasculature [2]. Although advances in understanding and treatment have improved survival rates, CDH remains to cause significant morbidity and mortality [3, 4]. Since lung morbidity can show a wide range of severity after CDH repair, periodic structured follow-up programs in specialised centres are strongly recommended to identify and treat the development of functionally significant deformities to extend the patients’ life expectancies [5, 6].

To assess pulmonary function in CDH patients and to quantify the regional distribution of ventilation (V), perfusion (Q), and their mismatch, scintigraphical measurements have been used in the past [7–10]. These studies demonstrated that lung perfusion is significantly reduced in the affected ipsilateral lung in comparison to the contralateral lung, and ventilation/perfusion (V/Q) mismatch continues to worsen after CDH repair, thereby potentially predicting long-term pulmonary morbidity [10, 11]. However, scintigraphical measurements lack morphological information and impose a radiation burden on patients. Subsequently, safety concerns regarding repeated radiation exposure and the need for children’s cooperation in the administration of radioactive gases have limited its application [11]. Other studies have suggested using MRI scans to evaluate the degree of structural damage and grade lung function morbidity without the need for ionising radiation [12–15]. With dynamic contrast-enhanced (DCE) MRI, quantitative lung perfusion parameters, such as pulmonary blood flow (PBF) and volume (PBV) can be measured in addition to morphological imaging during the same examination [2]. DCE-based studies have demonstrated observable perfusion differences between ipsilateral and contralateral lungs, which are reflected by the lower PBF and PBV values in the ipsilateral lung [2, 16, 17]. Although DCE-based studies have shown promising results, growing concerns about the safety of gadolinium‐based contrast agents and the inability to repeat measurements in cases of patient motion or technical difficulties limit the usefulness of DCE-based imaging [18].

As an alternative, non-contrast-enhanced MRI methods have been developed to evaluate lung function by exploiting periodic signal intensity changes associated with ventilation and perfusion [19–21]. Recently, these methods have been utilised to investigate lung function in adults [22], and in paediatric patients with cystic fibrosis [23–25]. However, these techniques have not been investigated in CDH patients.

The aims of this study are threefold. First, we investigate the feasibility of a non-contrast-enhanced MRI method for obtaining ventilation and perfusion values in 2-year-old patients after CDH repair. To this end, we use commonly available 2D balanced steady-state free precession (bSSFP) pulse sequences to acquire dynamic acquisitions together with a recently developed post-processing method called dynamic mode decomposition (DMD) to obtain pulmonary functional maps. Second, we investigate whether there are observable differences between the ipsilateral and contralateral sides with the DMD MRI method. Last, we identify whether there is a relationship in perfusion ratios of lungs between DMD-based perfusion imaging and DCE-based perfusion imaging.

## Materials and methods

### Patients

Fifteen patients after CDH repair were consecutively included between 2020 and 2023 as part of our local follow-up program, in which an MRI examination is included at the age of two years [26]. Detailed patient demographics are listed in Table S1 of supplementary materials. For all children, written informed consent was obtained from the parents. The study was approved by the local research ethics committee. The non-contrast-enhanced bSSFP protocols were added as supplementary sequences before the contrast-enhanced acquisitions, subject to feasibility and upon obtaining consent from the parents. Among the patient population, four were measured with Protocol A, two were measured with Protocol B, and nine were measured with both protocols (see below for protocol details).

### DMD-based functional pulmonary MRI

Non-contrast-enhanced functional lung imaging methods rely on dynamic acquisitions to capture signal variations arising from respiration and cardiac pulsation. To this end, imaging protocols with sufficiently high temporal resolution are employed during free-breathing to enable the observation of periodic signal variations. As such, ventilation- and perfusion-weighted information can be obtained without breathing manoeuvres or administration of contrast agents. Commonly, 2D bSSFP or gradient echo-based pulse sequences are used for rapidly acquiring lung images, and afterwards, lung structures are aligned using non-rigid registration software [27]. Subsequently, registered time-series images are analysed to obtain regional ventilation and perfusion information.

**Fig. 1:**
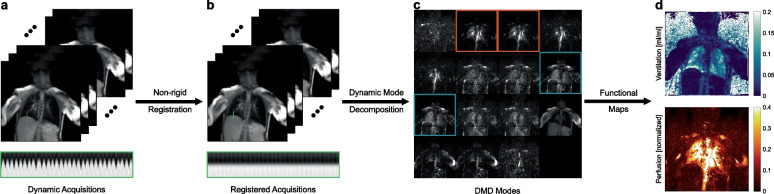
Workflow of the DMD MRI method. **a** Dynamic acquisitions are obtained during free-breathing to capture signal variations stemming from respiration and pulsation. **b** Lung structures are aligned using a non-rigid registration technique to enable the observation of regional parenchymal signal changes. Movements across measurements for a region (displayed with green line) before and after registration are also shown below acquisitions. **c** Registered time-series images are analysed using dynamic mode decomposition (DMD), and modes related to ventilation (displayed with blue boxes) and perfusion (displayed with red boxes) are identified. **d** Using these identified modes, ventilation and perfusion maps are calculated. Overall, functional maps can be obtained from dynamic acquisitions without the need for breathing manoeuvres or administration of contrast agents

In this work, a publicly available method based on DMD is utilised to analyse the time-series data to obtain functional maps robustly from dynamic acquisitions [28]. In essence, DMD is a data-driven modal decomposition algorithm capable of identifying dominant spatiotemporal structures latent within time-series data [29–31]. As such, DMD can determine the underlying oscillation frequencies and amplitudes in dynamic MR acquisitions, rendering it suitable for the identification of ventilation- and perfusion-related signal changes. With DMD MRI, dynamic MR images are analysed to identify ventilation and perfusion modes, and the ensuing fractional ventilation and normalised perfusion maps are calculated as previously explained [28]. The overall workflow is illustrated in Fig. 1.

### MRI protocols

MRI examinations were performed using a 1.5 T system (Magnetom Aera, Siemens Healthineers), with a combination of spine, head-neck, and body phased-array coils. Patients underwent free-breathing MRI scans in a supine position after an anaesthetist, who was present during the whole examination, have performed sedation using propofol. The lung function was assessed using DMD MRI and DCE-based perfusion imaging. For DMD MRI, dynamic images of the lung were acquired using bSSFP sequences adapted to accommodate paediatric patients with smaller field-of-view (FOV) and higher temporal resolution requirements; whereas for the DCE method, images were acquired using time-resolved angiography with stochastic trajectories (TWIST) sequence as previously described [2].

For non-contrast-enhanced imaging, two different bSSFP protocols (Table 1) were utilised to image at a single location. Protocol A was acquired using a larger FOV to improve the SNR characteristics and to maximise temporal resolution, whereas Protocol B was using a smaller FOV with a higher in-plane resolution, at the expense of SNR and temporal resolution. For both protocols, acquired slices were positioned posterior to the heart in the anterior–posterior dimension. The following parameters were common between protocols: flip angle α = 50º, pause between measurements = 0.07 s, phase partial Fourier = 6/8, parallel imaging with GRAPPA factor 3 using 24 integrated autocalibration lines. For both protocols, the initial six images were discarded due to transient state effects [19], and the remaining 274 images were analysed to obtain functional maps. Non-rigid image registration was performed using fMRLung 3.0 (Siemens Corporate Research) software [32, 33].

**Table 1:** Relevant parameter list of two different bSSFP protocols used for imaging

	**Protocol A**	**Protocol B**
FOV (mm × mm)	384 × 384	280 × 280
Voxel Resolution* (mm × mm)	1.5 × 1.5	1.1 × 1.1
Slice Thickness (mm)	12	12
TE/TR (ms/ms)	0.90/2.12	0.99/2.31
Acquisition Rate (images/s)	5.14	4.87

*TE*, echo time; *TR*, repetition time. *Voxel resolution with interpolation

DCE-based perfusion imaging was performed after the bSSFP acquisitions, using TWIST sequence with the following parameters: FOV = 416 × 416 mm^2^, voxel resolution = 1.9 × 1.9 mm^2^, slice thickness = 2 mm, TE/TR = 0.86/2.94 ms/ms, α = 30°, 56 slices per slab, view-sharing of 17% in the central and 20% in the outer region, parallel imaging with GRAPPA factor 2 in both phase encoding directions using 24 integrated autocalibration lines, temporal resolution of 1.5 s. Contrast agent (Dotarem, Guerbet) was administered after the acquisition of five baseline images, with a dosage of 0.05 mmol/kg body weight, diluted with the same volume of sodium chloride and followed by a sodium chloride bolus of 10 mL. Perfusion quantification was done using a deconvolution approach with an in-house certified OsiriX plugin [34] as previously described [17].

### Data analyses

To investigate image quality, quantitative analyses were performed on the magnitude images and functional maps. To this end, regions of interest (ROIs) containing lung parenchyma were manually segmented from magnitude images while excluding large vessels, and the quantitative metrics were calculated from the ROIs. The bSSFP protocols were compared using signal-to-noise ratio (SNR) and contrast-to-noise ratio (CNR) analyses. The SNR values were estimated on registered magnitude images based on the power spectrum of the time series [35], and frequencies above 2.2 Hz were considered noise. The CNR values were calculated on ventilation and perfusion maps and were defined as the ratio of mean functional amplitudes within the lung parenchyma and a homogeneous background region selected within liver tissue.

To investigate functional differences between lungs, ventilation and perfusion ratios between the ipsilateral and contralateral lungs (denoted as V_Ratio_ and Q_Ratio_ respectively) were calculated. For this purpose, mean functional values within the ROIs were obtained for each lung [36], and the ratios between the ipsilateral and contralateral lungs were calculated. Additionally, linear correlations and agreements between V_Ratio_ and Q_Ratio_ values obtained with Protocol A and Protocol B were assessed.

TWIST-based pulmonary perfusion was analysed by manually segmenting both lungs separately for each slice while leaving out the mediastinal structures. The arterial input function was derived from a ROI placed in the main stem of the pulmonary artery and the quantitative PBV and PBF parameters were calculated cumulatively from the segmented slices.

Lastly, DMD-based perfusion results were compared with TWIST-based PBF values to investigate the agreement between these methods. For this purpose, linear correlations, and agreements between TWIST-based perfusion ratio PBF_Ratio_ and DMD-based perfusion ratio values (Q_Ratio_) obtained with both protocols were assessed.

### Statistical analyses

For statistical analysis, all data were evaluated using the Kolmogorov–Smirnov test to check for normality. Subsequently, the differences in SNR, CNR, and functional ratios between Protocol A and Protocol B were compared using the Wilcoxon-Mann–Whitney test. The differences in DMD-based functional values between ipsilateral and contralateral lungs, as well as differences between DMD-based perfusion and TWIST-based perfusion ratios were compared using paired two-sided Wilcoxon signed rank tests. To assess linear correlations and agreements between functional ratios, Pearson’s correlation and Bland–Altman analyses were performed, where the biases were assessed using *t*-test. In all tests, $p<0.05$ was considered as statistically significant. The resulting correlation coefficient ($r$) was interpreted as: 0.00–0.19 negligible; 0.20–0.39 weak; 0.40–0.59 moderate; 0.60–0.79 strong; and 0.80–1.00 very strong correlation. All statistical analyses were performed using MATLAB (MathWorks).

## Results

### Feasibility of paediatric DMD MRI

**Fig. 2:**
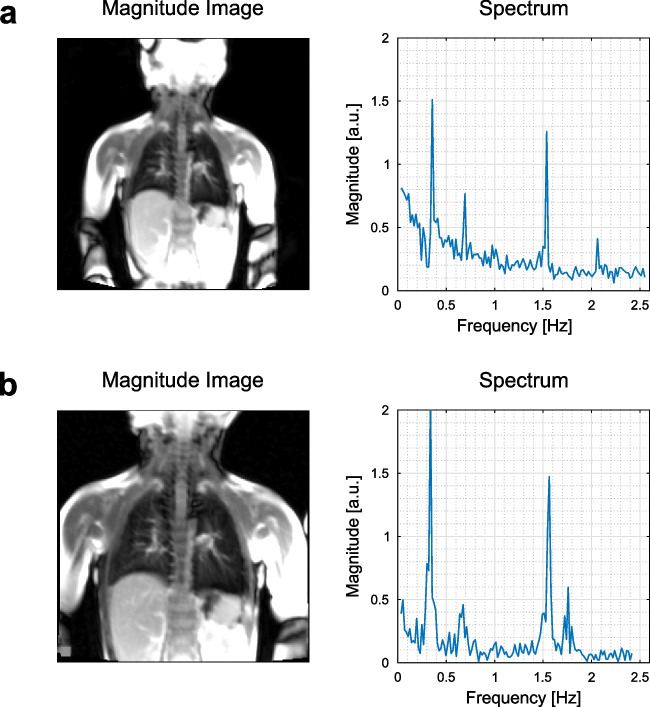
Exemplary magnitude images and frequency spectrums calculated from the acquisitions obtained with (**a**) Protocol A, and (**b**) Protocol B, from a CDH patient with left-sided hernia and ECMO treatment (patient no. 9). The magnitude images are scaled to better visualise lung parenchyma. Both protocols are able to successfully detect frequency peaks associated with respiration (~ 0.35 Hz) and cardiac pulsation (~ 1.55 Hz)

Representative cross-sections of bSSFP acquisitions obtained using both protocols are shown in Fig. 2 for a patient with left-sided herniation and extracorporeal membrane oxygenation therapy (ECMO) treatment. Additionally, frequency spectrums calculated from registered images using the mean signal intensity are also displayed. As illustrated, both protocols can distinguish frequency peaks associated with respiratory and cardiac cycles. The SNR and CNR values of protocols are listed in Tables 2 and 3 for Protocol A and Protocol B, respectively. As expected, Protocol A results in higher SNR values compared to Protocol B; nonetheless, CNR differences between the protocols are not significant ($p=0.86$ for CNR_Vent_, $p=0.73$ for CNR_Perf_).

**Table 2:** Quantitative measurements obtained from acquisition Protocol A. The average values are reported as mean ± standard deviation across patients

Patient No	SNR	CNR_Vent_	CNR_Perf_	V_Ratio_	Q_Ratio_
1	49.49 ± 17.32	7.52	2.07	0.63	0.62
2	60.22 ± 25.61	5.72	6.43	0.55	0.87
3	79.56 ± 37.62	16.26	8.35	0.73	0.82
4	31.49 ± 12.83	15.09	3.05	0.93	0.71
5	32.99 ± 14.49	14.73	5.32	0.57	0.75
6	32.04 ± 16.17	13.47	5.71	0.75	0.61
7	38.32 ± 13.63	14.56	9.46	0.94	1.38
8	25.14 ± 10.28	10.71	4.23	0.88	0.69
9	33.41 ± 11.56	12.27	5.84	0.93	0.65
10	33.47 ± 13.56	3.83	4.87	0.93	0.47
11	47.27 ± 16.74	10.06	5.40	0.85	0.56
12	47.43 ± 21.70	9.88	6.82	1.15	0.43
13	39.30 ± 12.86	7.08	5.05	1.11	0.60
Average	42.32 ± 14.71	10.86 ± 3.95	5.58 ± 1.97	0.84 ± 0.19	0.70 ± 0.24

*SNR*; signal-to-noise ratios, *CNR*_*Vent*_; contrast-to-noise ratio in ventilation maps; *CNR*_*Perf*_, contrast-to-noise ratio in perfusion maps; *V*_*Ratio*_, ipsilateral lung to contralateral lung ventilation ratios; *Q*_*Ratio*_ ipsilateral lung to contralateral lung perfusion ratios

**Table 3:** Quantitative measurements obtained from acquisition Protocol B. The average values are reported as mean ± standard deviation across patients

Patient No	SNR	CNR_Vent_	CNR_Perf_	V_Ratio_	Q_Ratio_
3	41.44 ± 17.42	18.63	3.92	0.70	0.83
5	24.05 ± 13.82	11.87	3.96	0.51	0.77
6	19.37 ± 11.01	13.39	5.27	0.75	0.70
7	27.01 ± 10.32	10.35	9.77	1.05	1.24
9	27.90 ± 12.43	11.75	5.25	0.95	0.54
10	33.13 ± 20.61	5.37	4.21	0.88	0.50
11	39.61 ± 16.54	5.44	5.71	0.79	0.64
12	35.34 ± 18.96	7.98	4.25	1.13	0.44
13	32.58 ± 12.28	9.48	8.70	0.95	0.57
14	36.13 ± 10.36	15.77	7.36	0.89	0.92
15	24.31 ± 11.75	16.07	4.41	1.02	0.71
Average	30.99 ± 6.99	11.46 ± 4.31	5.71 ± 2.02	0.88 ± 0.18	0.72 ± 0.23

*SNR*; signal-to-noise ratio, *CNR*_*Vent*_; contrast-to-noise ratio in ventilation maps; *CNR*_*Perf*_, contrast-to-noise ratio in perfusion maps; *V*_*Ratio*_, ipsilateral lung to contralateral lung ventilation ratios; *Q*_*Ratio*_ ipsilateral lung to contralateral lung perfusion ratios

**Fig. 3:**
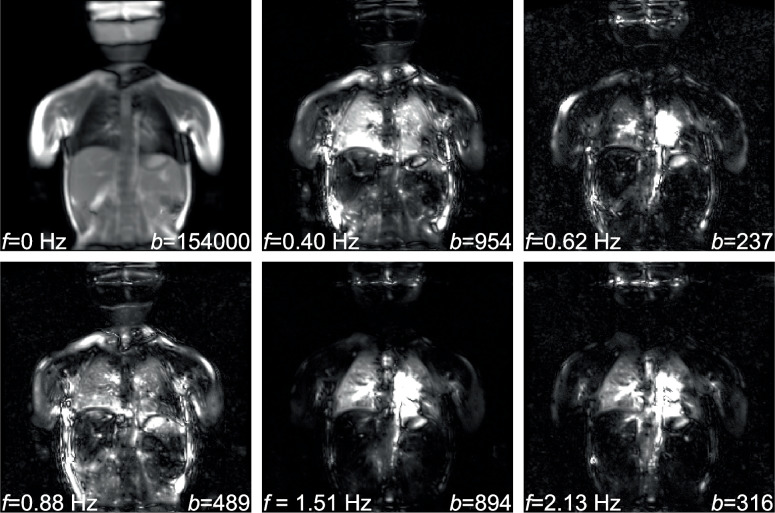
Exemplary dominant modes obtained with the DMD analysis from measurements of a patient with left-sided herniation and ECMO treatment (patient no. 3). Here, the dynamic images are obtained with Protocol A, and non-rigid registration is completed before the DMD analysis. The mode frequencies (*f*) and mode amplitudes (*b*) associated with the individual modes are also displayed overlaid on the modes. The DMD analysis identifies coherent spatiotemporal structures in the dynamic acquisitions, such as signal variations caused by respiration and pulsation, as well as secondary motions or harmonics. For this patient, modes related to pulmonary ventilation (mode with *f* = 0.40 Hz) and perfusion (mode with *f* = 1.51 Hz) were selected and used for the calculation of fractional ventilation and normalised perfusion maps

Figure 3 depicts exemplary modes obtained with the DMD from a patient measurement using Protocol A. Respective mode frequencies and mode amplitudes are also displayed overlaid on the modes. In our study cohort, ventilation frequencies ranged between 0.25 and 0.59 Hz, and perfusion frequencies ranged between 1.40 and 2.13 Hz. Overall, DMD analysis successfully identified dominant spatiotemporal features such as ventilation- and perfusion-related signal changes in all patients.

### DMD MRI-based pulmonary functional parameters

**Fig. 4:**
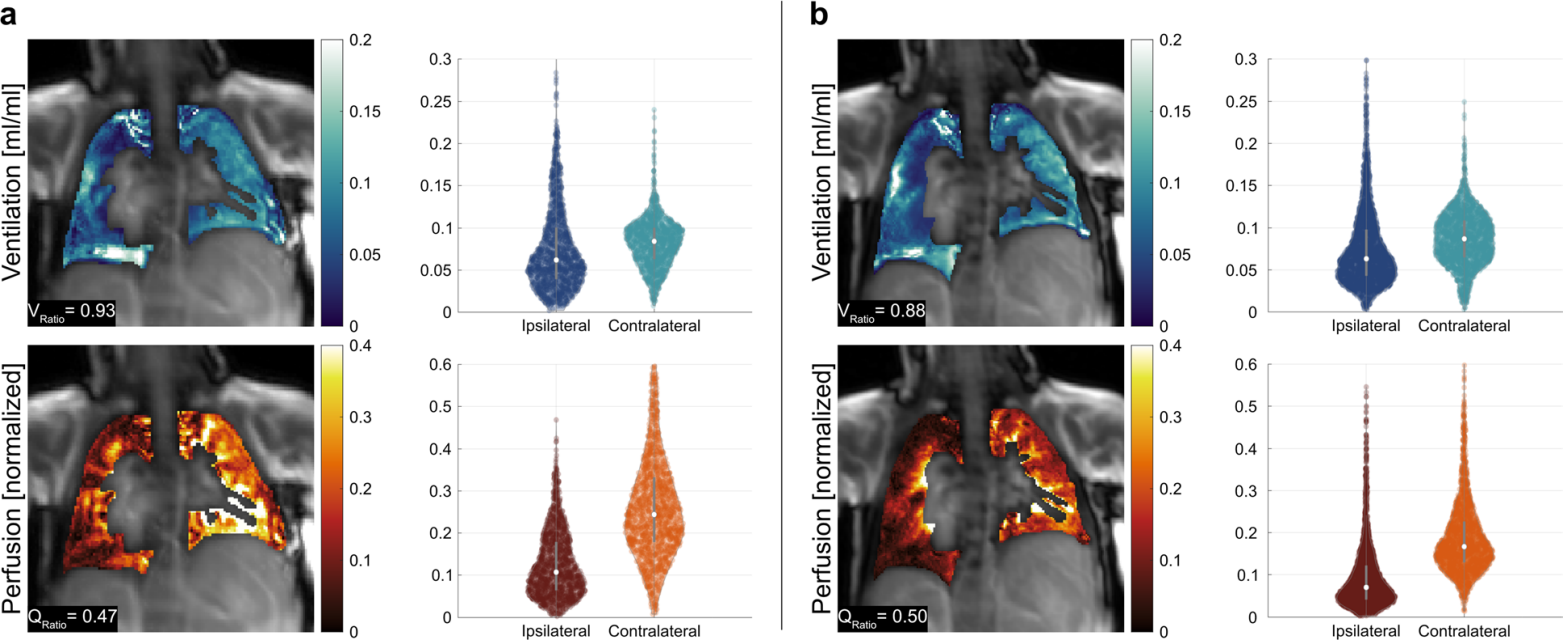
Exemplary fractional ventilation and normalised perfusion maps obtained through DMD MRI of a patient with right-sided herniation and without ECMO therapy (patient no. 10) obtained with (**a**) Protocol A, and (**b**) Protocol B. The maps are cropped to the region of interest for visualisation purposes. Next to the maps, violin plots depict the distribution of functional values of each lung. For both protocols, differences between ipsilateral and contralateral lungs are discernible in both ventilation and perfusion maps (V_Ratio_ and Q_Ratio_ are displayed overlaid on functional maps), as well as the accompanying violin plots, which imply impaired lung function in the ipsilateral lung relative to the contralateral lung

Illustrative ventilation and perfusion maps obtained from a patient are shown in Fig. 4 for both protocols. Additionally, violin plots of functional values are presented to illustrate the distribution differences between ipsilateral and contralateral lungs. For both protocols, functional maps are successfully obtained using DMD analysis and differences between ipsilateral and contralateral lungs are visible. Nevertheless, when the bSSFP protocols are compared, Protocol B displays finer structures owing to its higher spatial resolution.

The ipsilateral to contralateral ventilation (V_Ratio_) and perfusion (Q_Ratio_) ratios obtained with DMD MRI are reported in Tables 2 and 3. The mean fractional ventilation and normalised perfusion values obtained with DMD MRI for ipsilateral and contralateral lungs are reported in Tables S2 and S3 of the supplementary materials for Protocol A and B, respectively. On average, V_Ratio_ and Q_Ratio_ values are below 1, indicating reduced pulmonary functions in the ipsilateral lung compared to the contralateral lung. Specifically, the V_Ratio_ values for Protocol A and Protocol B are 0.84 ± 0.19 and 0.88 ± 0.18, respectively. Similarly, the Q_Ratio_ values for Protocol A and Protocol B are 0.70 ± 0.24 and 0.72 ± 0.23. Overall, our results demonstrate statistically significant reductions in ventilation ($p=0.01$ for Protocol A, $p=0.04$ for Protocol B) and perfusion ($p<0.01$ for both protocols) of the ipsilateral lungs with both protocols.

The Pearson’s correlation and Bland–Altman analyses of functional ratios obtained through DMD MRI with Protocol A and Protocol B are visualised in Figure S1 of the supplementary materials. For both V_Ratio_ and Q_Ratio_, we observe a very strong correlation ($r=0.92,p<0.01$ for ventilation ratios, and $r=0.97,p<0.01$ for perfusion ratios) and a close agreement between two protocols without a significant bias ($p>0.05$) or systematic difference.

### DCE-based TWIST pulmonary perfusion parameters

PBV and PBF parameters of ipsilateral and contralateral lungs as well as their ratios (PBV_Ratio_ and PBF_Ratio_) obtained through the TWIST method are reported in Table 4. Overall, the PBV_Ratio_ is 0.68 ± 0.19 and PBF_Ratio_ ratio is 0.68 ± 0.17, and both values indicate significantly lower perfusion ($p<0.01$) in the ipsilateral lungs.

**Table 4:** Perfusion parameters obtained through DCE-based TWIST protocol

**Patient No**	**PBV**_**Ipsi**_ [mL/100 mL]	**PBV**_**Cont**_ [mL/100 mL]	**PBV**_**Ratio**_	**PBF**_**Ipsi**_ [mL/100 mL/min]	**PBF**_**Cont**_ [mL/100 mL/min]	**PBF**_**Ratio**_
1	7.93 ± 4.76	13.95 ± 6.67	0.57	99.58 ± 54.97	175.57 ± 81.57	0.57
2	6.55 ± 3.85	8.67 ± 5.41	0.76	83.61 ± 45.24	110.90 ± 58.64	0.75
3	19.29 ± 16.40	19.56 ± 9.02	0.99	193.21 ± 89.47	250.67 ± 115.46	0.77
4	12.54 ± 8.15	26.76 ± 13.97	0.47	282.69 ± 185.67	390.96 ± 242.15	0.72
5	6.65 ± 6.77	13.10 ± 10.06	0.51	106.92 ± 101.01	176.95 ± 163.59	0.60
6	9.49 ± 5.85	16.40 ± 8.61	0.58	138.05 ± 77.94	234.64 ± 111.30	0.59
7	15.27 ± 5.99	13.15 ± 7.06	1.16	158.77 ± 66.57	131.78 ± 66.26	1.20
8	4.91 ± 3.90	9.64 ± 6.72	0.51	43.31 ± 36.40	95.19 ± 6.54	0.45
9	8.10 ± 5.10	11.85 ± 6.48	0.68	52.84 ± 35.05	84.94 ± 48.18	0.62
10	9.75 ± 6.74	13.59 ± 6.51	0.72	50.55 ± 34.85	83.38 ± 42.84	0.61
11	8.23 ± 5.30	12.86 ± 6.70	0.64	59.14 ± 38.75	102.06 ± 53.98	0.58
12	8.57 ± 4.88	13.73 ± 6.72	0.62	78.08 ± 45.39	133.47 ± 69.82	0.59
13	10.13 ± 7.32	16.94 ± 8.37	0.60	100.26 ± 55.57	163.72 ± 77.59	0.61
14	13.96 ± 5.67	17.08 ± 7.69	0.82	157.74 ± 65.67	195.50 ± 81.92	0.81
15	7.22 ± 4.26	12.06 ± 5.03	0.60	70.72 ± 40.23	99.24 ± 42.75	0.71
Average	9.91 ± 3.84	14.62 ± 4.40	0.68 ± 0.19	111.70 ± 65.17	161.93 ± 82.61	0.68 ± 0.17

*PBV*_*Ipsi*_, pulmonary blood volume in the ipsilateral lungs; *PBV*_*Cont*_, pulmonary blood volume in the contralateral lungs; *PBV*_*Ratio*_, ipsilateral lung to contralateral lung PBV ratios; *PBF*_*Ipsi*_, pulmonary blood volume in the ipsilateral lungs; *PBF*_*Cont*_, pulmonary blood volume in the contralateral lungs; *PBF*_*Ratio*_, ipsilateral lung to contralateral lung PBF ratios

### Comparisons between DMD-based perfusion and TWIST-based perfusion

**Fig. 5:**
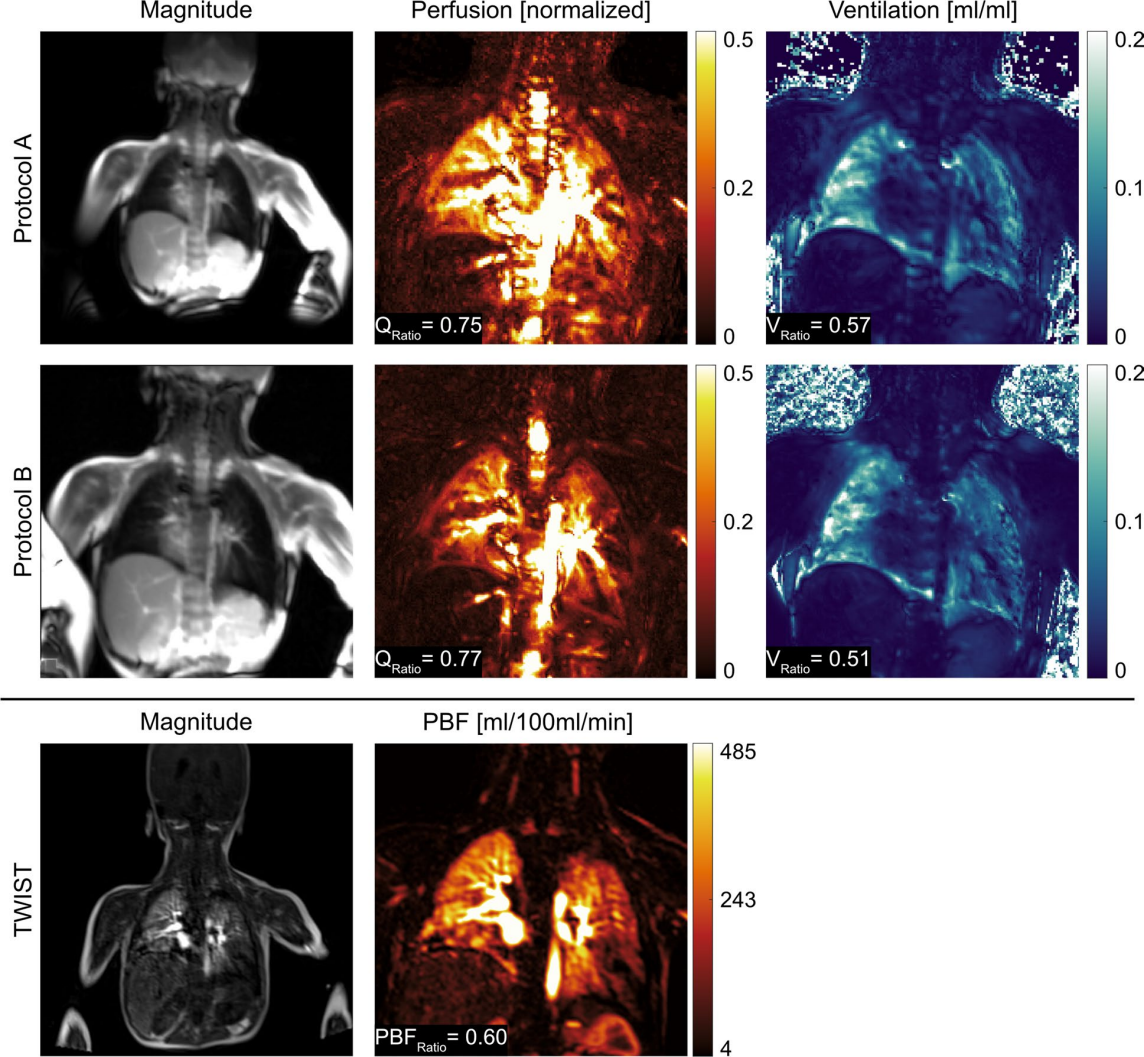
Representative magnitude images from bSSFP protocols, along with normalised perfusion, and fractional ventilation maps obtained through DMD MRI from a patient with left-sided hernia and ECMO therapy (patient no. 5). Additionally, a representative magnitude image and PBF map obtained from DCE-based TWIST protocol for the same patient from a similar slice location are displayed. For visualisation purposes, all functional maps are cropped to the region of interest. Overall, reductions in the ipsilateral lung can be observed visually, and can be confirmed by the resulting quantitative ratios (displayed overlaid on functional maps). Although the TWIST-based method can generate quantitative PBF maps, DMD MRI does not require the administration of contrast agents and is also capable of generating ventilation maps

A comparison of the DMD-based perfusion maps and the DCE-based TWIST perfusion map is provided in Fig. 5 for a patient with left-sided herniation with ECMO therapy. Additionally, ventilation maps obtained with DMD MRI are also displayed. For both methods, the reduction of perfusion values in the ipsilateral lung can be observed visually and quantitatively.

**Fig. 6:**
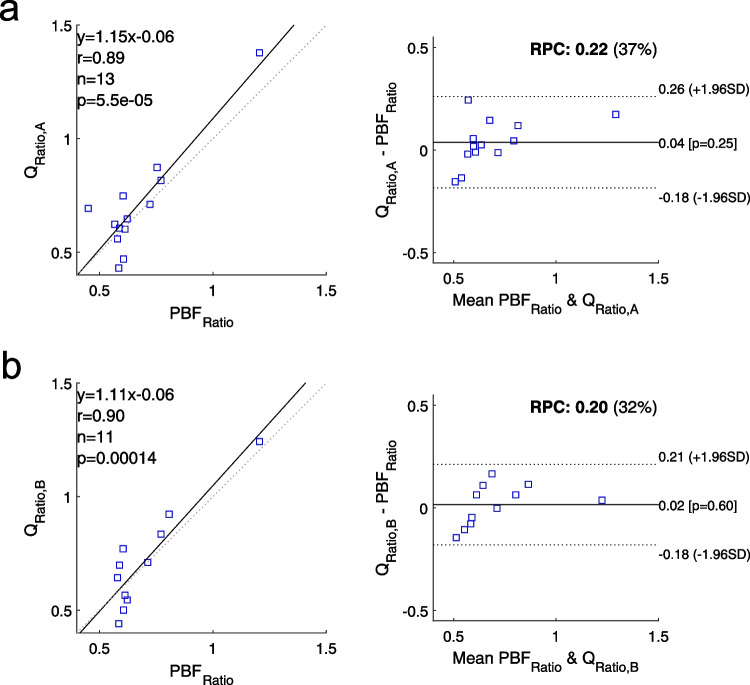
Pearson’s correlation and Bland–Altman analyses between perfusion ratios obtained via DCE-based TWIST (PBF_Ratio_) and DMD MRI (Q_Ratio_) for (**a**) Protocol A and (**b**) Protocol B. For both protocols, we observe a very strong correlation without any significant bias or systematic difference. (*r*, Pearson’s correlation coefficient; *RPC*, reproducibility coefficient.)

Pearson’s correlation and Bland–Altman analyses between Q_Ratio_ and PBF_Ratio_ are visualised in Fig. 6. Correlation coefficients are $r=0.89,p<0.01$ for Protocol A, and $r=0.90,p<0.01$ for Protocol B, indicating very strong correlations between Q_Ratio_ and PBF_Ratio_ for both protocols. Bland–Altman plots demonstrate a close agreement between Q_Ratio_ and PBF_Ratio_, with reproducibility coefficient (RPC) $RPC=0.22$ for Protocol A, and $RPC=0.20$ for Protocol B, and without a significant bias ($p>0.05$) or systematic difference for both protocols.

## Discussion

While the focus of most paediatric lung MRI studies centre on structural abnormalities, there is a growing utilisation of functional MRI techniques [37, 38]. Nonetheless, a crucial constraint arises from the faster cardiac and respiratory rates of paediatric patients [39], necessitating faster pulse sequences. Moreover, imaging paediatric patients require a reduced FOV, which limits the minimum achievable echo time [26], and thereby reduces the attainable SNR. Nevertheless, our results indicate that pulmonary functional imaging of 2-year-old CDH patients can be achieved at 1.5 T field strength using bSSFP protocols.

A key advantage of DMD MRI is its ability to generate both ventilation and perfusion maps from the same acquisition without any radiation burden or administration of intravenous contrast agents [2, 11]. Consequently, it can be repeated in cases where images are rendered non-diagnostic due to patient motion or technical difficulties [18]. Furthermore, it can facilitate contrast agent-free follow-up programs [26] and enable safer investigation of longitudinal trends in pulmonary functions [10]. Thus, it may improve our understanding of disease progression.

Our results indicate that both bSSFP protocols can acquire temporal features with success, and the trade-offs associated with Protocol B do not compromise the functional image quality. A possible explanation for the poorer CNR performance of Protocol A, despite its better SNR performance, is the partial volume effects, which may result in the blurring of features. Accordingly, we propose the use of Protocol B due to its higher in-plane resolution in future studies.

The values reported for V_Ratio_ are higher than perfusion ratios calculated between ipsilateral and contralateral lungs. Nevertheless, our results indicate that the ventilation differences between the ipsilateral and contralateral lungs are still significant. Additionally, ventilation of the ipsilateral lung seems to be less affected after CDH than perfusion, underlying the hypothesis that lung vessels are affected by CDH-associated lung hypoplasia.

Here, we were not able to compare the DMD MRI-based V_Ratio_ and Q_Ratio_ values with a healthy cohort, and the contralateral lungs were assumed to be relatively normal [9]. Previous studies with CDH patients have reported similar results when comparing the ipsilateral lung to the contralateral lung using scintigraphy [40, 41], SPECT [9, 11], and DCE MRI [2, 16, 17]. The V_Ratio_ and Q_Ratio_ results reported in this study are in line with those observations. Additionally, while a gold standard was not available for ventilation maps; we have observed a very strong agreement between Q_Ratio_ and PBF_Ratio_ values obtained from the same patients.

In the current study, the bSSFP acquisitions were limited to a single slice position, and in some patients, acquisitions with both bSSFP protocols were not possible due to scan time constraints. In future work, multiple slice locations could be acquired using a single protocol to increase lung volume coverage. Additionally, thinner slices may be required to offset the partial volume effects at the expense of SNR. To this end, recent developments incorporating low-rank priors during image reconstruction [42] showed promising results in functional lung imaging [43]. Another limitation of the study arises from the inherent issues associated with the quantification methods [44]; which creates a dependency on the underlying image contrast. Nevertheless, the effects of the image contrast are cancelled out when the ratio of the lungs is calculated, and therefore, it does not hinder the observation of functional differences between the ipsilateral and contralateral lungs. With improved quantification methods, DMD MRI has the potential for analysing quantitative lung parameters and V/Q mismatch associated with CDH [7, 8, 10, 45]; meanwhile, assessing defect percentage metrics may offer further insights [47]. Recent studies demonstrated that functional maps obtained via conventional bSSFP acquisitions suffer from banding artefacts, resulting in inhomogeneous ventilation maps [44]. To improve robustness against magnetic field inhomogeneities, advanced acquisition techniques can be employed [44, 46]. Lastly, our study was limited to a small sample size. While further investigations with larger cohorts are warranted, these preliminary findings indicate DMD MRI as a promising option for functional lung imaging.

In summary, we have successfully demonstrated the ability to measure pulmonary functional information in 2-year-old CDH patients. Our approach involved analysing free-breathing non-contrast-enhanced dynamic bSSFP acquisitions through DMD to obtain ventilation and perfusion maps. Our results are in line with previously reported values obtained with different modalities. Moreover, DMD-based perfusion results demonstrate a very strong agreement with the perfusion results obtained through the DCE-based TWIST method. Overall, DMD MRI is a promising tool for the analysis of pulmonary function in paediatric CDH patients and holds potential for longitudinal assessments of pulmonary functional changes.

### Supplementary information

Below is the link to the electronic supplementary material.

Supplementary file1 (PDF 279 KB)

## Abbreviations

bSSFP: Balanced steady-state free precession
CDH: Congenital diaphragmatic hernia
CNR: Contrast-to-noise ratio
DCE: Dynamic contrast-enhanced
DMD: Dynamic mode decomposition
ECMO: Extracorporeal membrane oxygenation therapy
FOV: Field-of-view
GRAPPA: Generalized autocalibrating partially parallel acquisition
MRI: Magnetic resonance imaging
PBF: Pulmonary blood flow
PBV: Pulmonary blood volume
Q: Pulmonary perfusion
ROI: Region of interest
RPC: Reproducibility coefficient
SNR: Signal-to-noise ratio
TE: Echo time
TR: Repetition time
TWIST: Time-resolved angiography with stochastic trajectories
V: Pulmonary ventilation

## Declarations

### Guarantor

The scientific guarantor of this publication is Dr. Med. Meike Weis.

### Statistics and biometry

One of the authors has significant statistical expertise.

### Study subjects or cohorts overlap

No study subjects or cohorts have been previously reported.

### Methodology

• prospective

• performed at one institution
