# Supplementary material for: Functional lung imaging of 2-year-old children after congenital diaphragmatic hernia repair using dynamic mode decomposition MRI
Source: Eur Radiol. 2023 Nov 8;34(6):3761–72. doi: 10.1007/s00330-023-10335-6 (PMC11166761; doi:10.1007/s00330-023-10335-6)
Supplement: Supplementary file 1 — Supplementary file1 (PDF 279 KB) [file 330_2023_10335_MOESM1_ESM.pdf]

Supplementary Materials

Eur Radiol (2023) Ilicak E, Thater G, Ozdemir S et al

Table S1: Patient demographics.

Patient No.	CDH Location	ECMO Status / Duration (days)	Protocol	Sex	Age (weeks)	Weight (kg)	Height (cm)
1	Left	No / –	A	Female	101	11.4	84
2	Left	Yes / 11	A	Female	104	7.7	80
3	Left	Yes / 8	A & B	Male	115	19	106
4	Right	Yes / 8	A	Female	108	9.5	85
5	Left	Yes / 10	A & B	Male	105	12.8	86
6	Left	Yes / 10	A & B	Female	109	11.6	83
7	Left	No / –	A & B	Female	107	11.7	93
8	Left	Yes / 9	A	Female	109	10	92
9	Left	Yes / 9	A & B	Male	106	15	93
10	Right	No / –	A & B	Female	103	10.3	83
11	Left	No / –	A & B	Female	103	9.6	83
12	Left	No / –	A & B	Female	116	11	85
13	Right	Yes / 9	A & B	Male	117	10	68
14	Left	No / –	B	Female	109	13.5	120
15	Right	Yes / 10	B	Male	105	10.7	88

CDH, Congenital diaphragmatic hernia; *ECMO,* Extracorporeal membrane oxygenation therapy. Patch implantations were required in all patients.

Table S2: Mean fractional ventilation (V, scaled by a factor of 100 for better visualization) and normalized perfusion (Q) results calculated over ipsilateral (V_{Ipsi}, Q_{Ipsi}) and contralateral (V_{Cont}, Q_{Cont}) lungs from images obtained with Protocol A. Their ratios (V_{Ratio} and Q_{Ratio}) are also reported. The average values are reported as mean ± standard deviation across patients.

**Patient No.**	V_{Ipsi} [ml/ml×100]	V_{Cont} [ml/ml×100]	V_{Ratio}	Q_{Ipsi} [normalized]	Q_{Cont} [normalized]	Q_{Ratio}
1	6.40±5.27	10.17±3.93	0.63	0.11±0.07	0.17±0.10	0.62
2	1.85±1.30	3.37±2.48	0.55	0.27±0.23	0.31±0.19	0.87
3	5.12±2.59	7.02±3.39	0.73	0.26±0.20	0.32±0.14	0.82
4	5.28±2.98	5.65±2.16	0.93	0.24±0.13	0.34±0.25	0.71
5	6.09±2.92	10.75±3.99	0.57	0.27±0.16	0.36±0.16	0.75
6	4.98±2.33	6.63±2.91	0.75	0.27±0.15	0.45±0.13	0.61
7	5.11±2.15	5.45±3.28	0.94	0.35±0.16	0.25±0.13	1.38
8	7.03±3.22	7.99±3.84	0.88	0.24±0.15	0.34±0.17	0.69
9	7.54±4.21	8.09±4.39	0.93	0.37±0.20	0.56±0.19	0.65
10	7.68±5.54	8.25±2.93	0.93	0.13±0.08	0.27±0.14	0.47
11	7.28±3.01	8.53±4.67	0.85	0.26±0.19	0.46±0.15	0.56
12	5.56±2.41	4.85±2.55	1.15	0.22±0.15	0.50±0.20	0.43
13	6.01±2.35	5.39±2.03	1.11	0.38±0.17	0.63±0.21	0.60
Average	5.84±1.54	7.09±2.14	0.84±0.19	0.26±0.08	0.38±0.13	0.70±0.24

Table S3: Mean fractional ventilation (V, scaled by a factor of 100 for better visualization) and normalized perfusion (Q) results calculated over ipsilateral (V_{Ipsi}, Q_{Ipsi}) and contralateral (V_{Cont}, Q_{Cont}) lungs from images obtained with Protocol B. Their ratios (V_{Ratio} and Q_{Ratio}) are also reported. The average values are reported as mean ± standard deviation across patients.

**Patient No.**	V_{Ipsi} [ml/ml×100]	V_{Cont} [ml/ml×100]	V_{Ratio}	Q_{Ipsi} [normalized]	Q_{Cont} [normalized]	Q_{Ratio}
3	4.81±2.57	6.84±3.52	0.70	0.13±0.11	0.16±0.06	0.83
5	5.48±2.44	10.72±4.25	0.51	0.15±0.09	0.19±0.11	0.77
6	8.07±3.73	10.82±5.39	0.75	0.18±0.11	0.26±0.10	0.70
7	8.05±3.55	7.65±5.10	1.05	0.22±0.11	0.17±0.09	1.24
9	7.50±4.16	7.87±4.09	0.95	0.22±0.13	0.41±0.16	0.54
10	7.66±4.92	8.69±3.19	0.88	0.09±0.08	0.19±0.09	0.50
11	13.75±5.98	17.46±6.61	0.79	0.19±0.19	0.29±0.10	0.64
12	8.05±3.65	7.11±3.67	1.13	0.15±0.12	0.33±0.13	0.44
13	10.05±4.59	10.56±3.56	0.95	0.26±0.13	0.45±0.18	0.57
14	5.69±2.08	6.37±2.69	0.89	0.21±0.18	0.22±0.09	0.92
15	8.74±4.13	8.58±4.63	1.02	0.14±0.10	0.20±0.16	0.71
Average	7.98±2.45	9.33±3.12	0.88±0.18	0.18±0.05	0.26±0.10	0.72±0.23

Figure S1: Pearson's correlation and Bland-Altman analyses of functional ratios obtained through DMD MRI with Protocol A and Protocol B. V_{Ratio} and Q_{Ratio} values obtained with two non-contrast-enhanced bSSFP protocols display a very strong correlation and a close agreement with each other without any systematic differences.

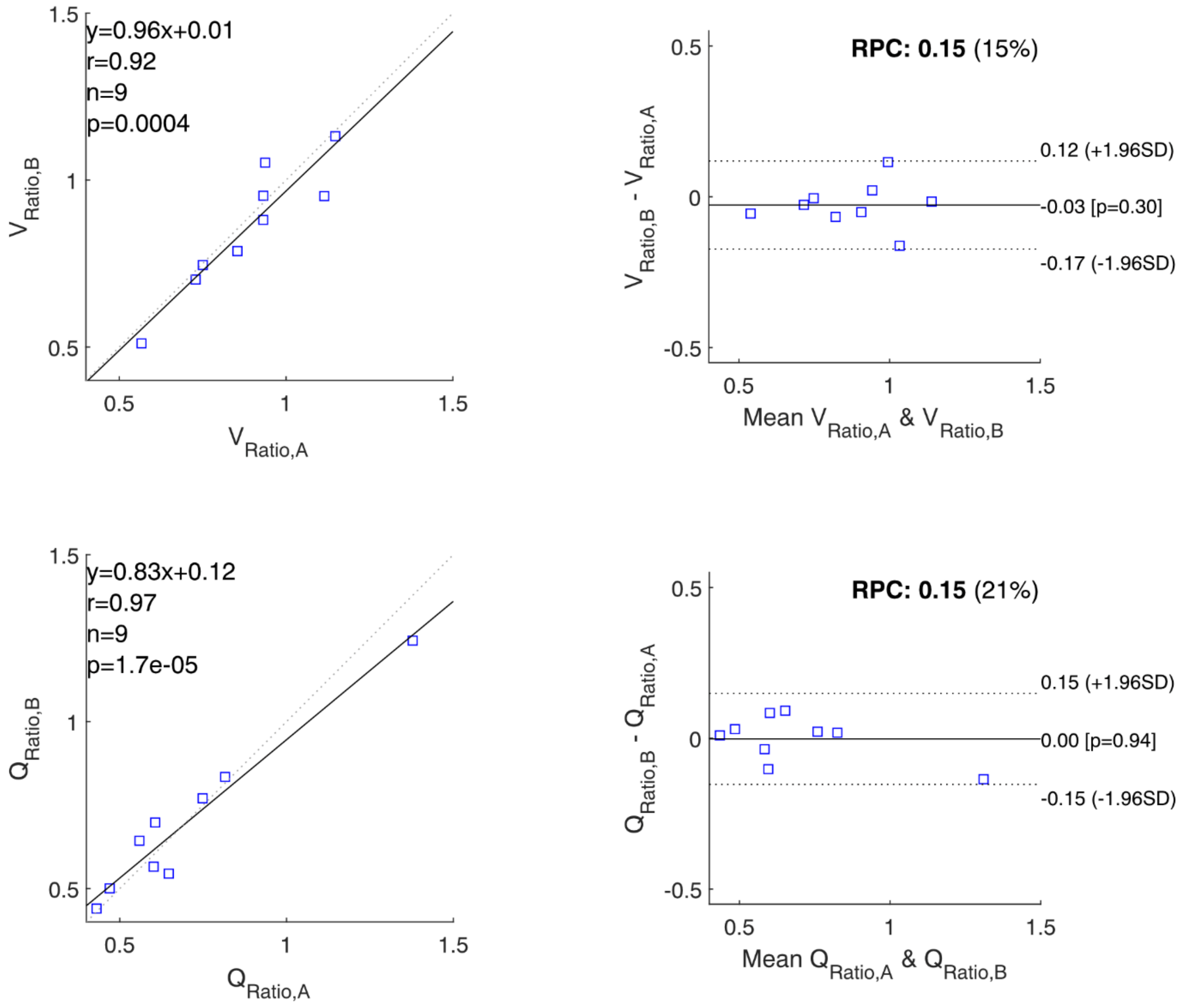

$V_{Ratio,A}$, ipsilateral to contralateral lung ventilation ratios obtained with Protocol A; $V_{Ratio,B}$, ipsilateral to contralateral lung ventilation ratios obtained with Protocol B; $Q_{Ratio,A}$ ipsilateral lung to contralateral lung perfusion ratios obtained with Protocol A; $Q_{Ratio,B}$ ipsilateral lung to contralateral lung perfusion ratios obtained with Protocol B; *r*, Pearson's correlation coefficient; *RPC*, reproducibility coefficient.
